# Supplementary material for: Chemical Composition of Essential Oil from Citrus reticulata Blanco cv. Chachiensis (Chachi) and Its Anti-Mosquito Activity against Pyrethroid-Resistant Aedes albopictus
Source: Insects. 2024 May 11;15(5):345. doi: 10.3390/insects15050345 (PMC11122156; doi:10.3390/insects15050345)
Supplement: Supplementary file 1 [file insects-15-00345-s001.zip › insects-2930791-supplementary.pdf]

Chemical Composition of Essential Oil from *Citrus reticulata* Blanco cv. Chachiensis (Chachi) and its Anti-mosquito Activity against Pyrethroid-resistant *Aedes albopictus*

Jifan Cao [1,2], Wende Zheng [1,2], Baizhong Chen [3], Zhenping Yan [1,2], Xiaowen Tang [1,2], Jiahao Li [1,2], Zhen Zhang [1,2], Song Ang [1,2], Chen Li [1,2], Rihui Wu [1,2], Panpan Wu [1,2,*] and Wen-Hua Chen [1,2,*]

[1] School of Pharmacy and Food Engineering, Wuyi University, Jiangmen 529020, P. R. China

[2] International Healthcare Innovation Institute (Jiangmen), Jiangmen 529040, P. R. China

[3] Guangdong Xinbaotang Biotechnology Co. Ltd, Jiangmen 529100, P. R. China

Supporting Information

Table S1. ANOVA one way test of acetylcholinesterase (AChE) inhibition

	Sum of Squares	df	F	*p*
Between Groups	2100	5	188.1	<0.0001
Within Groups	26.79	12		
Total	2126	17		

Table S2. ANOVA one way test of carboxylesterase (EST) inhibition

	Sum of Squares	df	F	*p*
Between Groups	2338	6	43.75	<0.0001
Within Groups	124.7	14		
Total	2463	20		

Table S3. ANOVA one way test of glutathione-S-transferase (GST) inhibition

	Sum of Squares	df	F	*p*
Between Groups	2447	6	41.55	<0.0001
Within Groups	137.4	14		
Total	2584	20		

Table S4. ANOVA one way test of multifunctional oxidase (MFO) inhibition

	Sum of Squares	df	F	*p*
Between Groups	1959	6	258.8	<0.0001
Within Groups	17.66	14		
Total	1976	20		

Table S5. Carboxylesterase (EST), glutathione-S-transferase (GST) and multifunctional oxidase (MFO) activities in larvae and adult mosquitoes of susceptible and resistant starin of *Aedes albopictus*

A.albopictus	Starin	EST ($nmol.min^{-1}.mg^{-1}$)	GST ($nmol.min^{-1}.mg^{-1}$)	MFO ($nmol.min^{-1}.mg^{-1}$)
Larvae	Lab-S	12.21 ± 3.11	1.95 ± 1.43	0.02 ± 0.00
	Pyr-R	11.10 ± 2.60	7.05 ± 1.14	1.72 ± 0.20
Adult	Lab-S	15.69 ± 3.27	8.66 ± 2.53	0.97 ± 0.09
	Pyr-R	21.28 ± 5.40	11.50 ± 1.90	1.21 ± 0.12

Table S6. ANOVA one way test of Shannon

	Sum of Squares	df	F	*p*
Between Groups	83.11	5	23.84	<0.0001
Within Groups	8.368	12		
Total	91.48	17		

Table S7. ANOVA one way test of Simpson

	Sum of Squares	df	F	*p*
Between Groups	0.3566	5	18.77	<0.0001
Within Groups	0.0456	12		
Total	0.4022	17		

Table S8. ANOVA one way test of Chao 1

	Sum of Squares	df	F	*p*
Between Groups	2466811	5	197.0	<0.0001
Within Groups	30047	12		
Total	2496858	17		

Table S9. ANOVA one way test of the relative abundance of *Wolbachia*

	Sum of Squares	df	F	*p*
Between Groups	4878	5	184.5	<0.0001
Within Groups	63.45	12		
Total	4942	17		

Table S10. ANOVA one way test of the relative abundance of *Enterobacter*

	Sum of Squares	df	F	*p*
Between Groups	1942	5	36.75	<0.0001
Within Groups	126.8	12		
Total	2069	17		

Table S11. ANOVA one way test of the relative abundance of *Elizabethkingia*

	Sum of Squares	df	F	*p*
Between Groups	558.3	5	25.56	<0.0001
Within Groups	52.42	12		
Total	610.7	17		

Table S12. ANOVA one way test of the relative abundance of *Pseudomonas*

	Sum of Squares	df	F	*p*
Between Groups	193.8	5	224.5	<0.0001
Within Groups	2.072	12		
Total	195.9	17		
